# Comparison of Monocyte Distribution Width (MDW) and Procalcitonin for early recognition of sepsis

**DOI:** 10.1371/journal.pone.0227300

**Published:** 2020-01-10

**Authors:** Ennio Polilli, Federica Sozio, Antonella Frattari, Laura Persichitti, Marina Sensi, Raffaella Posata, Marco Di Gregorio, Antonina Sciacca, Maria Elena Flacco, Lamberto Manzoli, Giancarlo Di Iorio, Giustino Parruti

**Affiliations:** 1 Clinical Pathology Unit, Pescara General Hospital, Pescara, Italy; 2 Infectious Diseases Unit, Pescara General Hospital, Pescara, Italy; 3 Unit of Intensive Care, Pescara General Hospital, Pescara, Italy; 4 Clinical Pathology Department, University of Chieti, Chieti, Italy; 5 Local Health Unit of Pescara, Pescara, Italy; 6 Department of Medical Sciences, University of Ferrara, Ferrara, Italy; National Yang-Ming University, TAIWAN

## Abstract

We carried out a prospective observational study to evaluate whether Monocyte Distribution Width (MDW) may play a role in identifying patients with sepsis in comparison with Procalcitonin (PCT). We prospectively enrolled all consecutive patients hospitalized at the Infectious Diseases Unit of Pescara General Hospital for bacterial infection or sepsis. MDW values were collected for all patients. Clinical characteristics, demographic data, past and present medical history, microbiological results, PCT, as well as neutrophil and monocytes indices at entry were compared in the 2 groups. Two-hundred-sixty patients were enrolled, 63.5% males, aged 59.1±19.5 years. Sepsis was diagnosed in 105 (40.4%); in 60 (57.1%) at least 1 microorganism was isolated from blood cultures. In multivariate models, MDW as a continuous variable (OR:1.57 for each unit increase; 95%CI: 1.31–1.87, p<0.001) and PCT˃1 ng/mL (OR: 48.5; 95%CI: 14.7–160.1, p<0.001) were independently associated with sepsis. Statistical best cut points associated with sepsis were 22.0 for MDW and 1.0 ng/mL for PCT whereas MDW values<20 were invariably associated with negative blood cultures. At ROC curve analysis, the AUC of MDW (0.87) was nearly overlapping that of PCT (0.88). Our data suggest that incorporating MDW within current routine WBC counts and indices may be of remarkable use for detection of sepsis. Further research is warranted.

## Background

Sepsis and septic shock are increasingly reported as a major cause of morbidity and mortality, especially among comorbid and hospitalized patients [1,2]. Survival to septic episodes is significantly improved after early recognition of sepsis and sepsis-related organ dysfunction and early start of appropriate causative and supportive treatment [1,2]. As a consequence, the search for diagnostic tools that may ease diagnosis of sepsis and quick evaluation of sepsis-related disease severity is intensive, as sepsis is a heterogeneous and elusive condition with highly variable and non-specific symptoms and signs [2,3]. In recent years, Procalcitonin (PCT) rose to the recognized status of best tool to evaluate ensuing sepsis in at risk patients, as its increase usually precedes septic shock in bacteremic patients [2,4]. In particular, serial evaluations of PCT may be sensitive to drive appropriate empiric treatment. The major limitations of PCT monitoring are represented by costs linked to repeated assaying, as well as by false negative results in patients with invasive fungal infections [5]. Hematological biomarkers, such as blood cell indices, have been explored in late years for their potential role in predicting ensuing septic episodes in at risk patients [6–8]. In particular, preliminary evidence was collected on the possible relevance of Monocyte Distribution Width (MDW) [9]. It has been demonstrated that monocytes increase their size upon activation in bacteremic patients, and that this infection-related variation in size may be easily monitored by measuring the spread of monocytes in coulter chambers [9]. As of yet, however, in spite of such recognized experimental potential, the use of MDW in clinical practice is yet lagging behind. The aim of the present analysis was to evaluate whether blood cell counts and monocyte volume measures can have a substantial role in identifying patients with bacteremia and sepsis among those hospitalized an Infectious Disease Unit for suspected infection.

## Patients and methods

We performed an observational prospective, monocentric cohort study to evaluate the possible association of population cell data parameters with ensuing sepsis at the Infectious Diseases Unit of Pescara General Hospital. The study was conducted in accordance with the amended Declaration of Helsinki. The local Health Administrative Board in Pescara reviewed in detail the study plan, set up by the Infectious Diseases Staff in Pescara General Hospital. Informed consent was not required, because confidentiality was guaranteed and no interventions were performed beyond ordinary good and standard clinical practices (measurement of blood cell volumes and indices). Written informed consent, however, was provided by all patients upon Hospital admission for use of anonymized clinical and laboratory data for Institutional research purposes. The study was started in August, 2017 and ended in September, 2018. Patients were consecutively enrolled whenever hospitalized for suspected infection or sepsis. Clinical characteristics of patients at entry, including demographics (age and gender); vital signs (heart rate, respiratory rate, body temperature, blood pressure), past medical history and other laboratory examinations (white blood cell counts, blood gas analysis, blood biochemistry) were prospectively collected. Patients with bacterial infections and underling conditions potentially associated with dysregulation of their immune system (AIDS, organ or bone marrow transplantation, malignancy, hematologic diseases) were excluded from further evaluation. Previous investigations indicated that factors of progression into sepsis in infected patients include age, gender, immune status and underlying chronic diseases and comorbidities that may play a major role in facilitating organ dysfunction [10, 11]. The Charlson Comorbidity Index (CCI) was therefore calculated for each patient at hospital entry, using criteria defined by Charlson et al. (1987) [12,13], including sixteen disease conditions with different weights: Myocardial Infarction, Congestive Heart Failure, Peripheral Vascular disease, Cerebrovascular disease, Dementia, COPD (Chronic Obstructive Pulmonary Disease), Connective Tissue diseases, Peptic Ulcer disease, Diabetes Mellitus, Moderate to severe CKD (Chronic Kidney Disease), Hemiplegia, Leukemia, Malignant Lymphoma, Solid Tumor, Liver disease, AIDS.

Sepsis was defined as life-threatening organ dysfunction caused by a dysregulated host response to infection as reported in The Third International Consensus Definitions for Sepsis and Septic Shock (Sepsis-3) [1]. Organ dysfunction was identified as an acute change in total SOFA (Sequential Organ Failure Assessment) score ≥2 points consequent to the infection. Patients with septic shock were identified with a clinical construct of sepsis with persisting hypotension requiring vasopressors to maintain MAP (Mean Arterial Pressure) ≥65 mm Hg and having a serum lactate level >2 mmol/L (18 mg/dL) despite adequate volume resuscitation [1]. We showed qSOFA (quick Sequential Organ Failure Assessment) in univariate analyses because qSOFA was the recommended score by sepsis 3 outside the ICU (Intensive Care Unit), as SOFA requires multiple laboratory tests and may be cumbersome outside the ICU. qSOFA, however, lacks of sensitivity [14–16] and for this reason we also retrieved SOFA scores, retrospectively calculated.

Blood cell counts (Red Blood Cells, RBC; Hemoglobin, HGB; Hematocrit, HCT; Mean Cell Volume, MCV; Mean Cell Hemoglobin Concentration, MCHC; Mean Platelet Volume, MPV; Platelet Cell Width, PDW; Red Cell Distribution Width, RDW) and platelet indices were obtained with the UniCel DxH 800 instrument (Beckman Coulter, Inc, Brea, California). Mean Neutrophil Volume (MNV), Neutrophil Distribution Width (NDW), Mean Monocyte Volume (MMV), and Monocyte Distribution Width (MDW) were measured as was described in Celik et al. (2012) [17]. Briefly, they are estimated in the adult population utilizing Volume, Conductivity, and Scatter (VCS) technologies [17, 18]. VCS parameters can detect morphologic changes in immature and reactive cells, similarly to microscopic evaluation of a peripheral blood smear [17–19]. Considered VCS parameters [Mean Neutrophil Volume (MNV), Neutrophil Distribution Width (NDW), Mean Monocyte Volume (MMV), and Monocyte Distribution Width (MDW)] were analyzed on the first blood sample at hospital entry, their values were omitted in medical report because they were performed for research purposes only and were retrieved for statistical analyses. Values of MDW and PCT included in our statistical analyses were therefore estimated and paired at hospital entry.

Complete Blood Counts (CBC) and all Cell Population Data including MNV, NDW, MMV and MDW determinations were analyzed with hematologic analyzer UniCel DxH 800 (Beckman Coulter, Inc, Brea, California). All determinations of MNV, NDW, MMV and MDW were measured from a K3EDTA whole-blood venous sample within 2 hours of collection and were performed in the same tubes of blood used for CBC determinations. Turnaround Time for such measurements was within the time of Complete Blood Counts. Quality control was performed by monitoring performances of diagnostic processes using commercial controls. Controls with known characteristics were analyzed daily in the same way as samples, and results of the analyzed controls were compared with standard characteristics using statistical methods calculated by the same instrument. Quality control of CBC and of cell population data including MNV, NDW, MMV and MDW were performed daily with COULTER® 6C Cell Control, enabling monitoring of system performance for all directly measured and calculated parameters, and with COULTER LATRON CP-X Control, a suspension of stable polystyrene particles of uniform size with a diameter CV ≤3.0%. Latron CP-X was used as part of the daily quality control procedure, to monitor the stability of the electrical processing and the fluidic flow rate systems used to measure the volume, conductivity and light scattering characteristics of cells as they pass through the flow cell. COULTER S-CAL Calibrator was used in the UniCel DxH 800 to determine the calibration factors for directly measured CBC parameters; differential blood counts were not required. MNV, NDW, MMV and MDW parameters were derived from monocytes and neutrophils, and use of a calibrator was not required. Producers of hematological analyzers do not provide any unit for MDW and other positional parameters, as previously published [20, 21]. Positional parameters derive from an algorithmic application that transforms femtoliters in positions on the x-axis of the scatterplot, of entity proportionate to the value of the cell volume.

Microbiological isolates, including MRSA (Methicillin-Resistant *Staphylococcus Aureus*) and any other MDROs (Multi-Drug Resistant Organisms), were retrieved and classified as in Siegel et al. (2007) [22]. We collected all the information on the sequential antibiotic regimens prescribed, other supportive therapies and length of hospitalization in days.

Sensitivity and specificity of MDW were compared with those of procalcitonin, the most important and validated laboratory marker associated with sepsis in clinical practice, mainly used for antibiotic management of systemic infections. MDW and PCT measurements were performed at hospital entry at the same time of the first determination of CBC and biochemical parameters. Blood for PCT measurement was collected in tubes with Lithium Heparin anticoagulant and was analyzed by Elecsys® BRAHMS Procalcitonin (Roche Diagnostics GmbH, Mannheim, Germany), an automatic analyzer that measures PCT concentration in the blood with a Sandwich ELISA procedure. All procedures were performed following the manufacturer's instructions.

ROC curve analysis for the prediction of sepsis was performed in order to compare the AUC curves of MDW and PCT. Level of statistical significance for differences between AUCs were cal-culated as described by De Long et al. (1988) [23]. Sensitivity, specificity, PPV (positive predictive value) and NPV (negative predictive value) with their 95%CI for different cut points of MDW and PCT for sepsis prediction were calculated. Best statistical cut points for MDW and PCT were estimated using the classical Youden method [24].

Previous studies established that values of procalcitonin below of 0.5 ng/mL were unlikely correlated with progression of infection. Values between 0.5 ng/mL and 1 ng/mL turned out probably related with progression of infection with poor specificity. Values higher than 1 ng/mL were very likely linked to progression of infection and for this reason we choose to dichotomize PCT as a binary variable with a cut-off of 1 ng/mL and presented our results as multivariate analyses [25].

Differences in the selected variables were first examined using the chi-squared test for categorical variables and the non-parametric Kruskal-Wallis rank test for continuous variables. Stepwise forward logistic regression was used to examine the independent association between sepsis and each potential determinant. Statistical significance was defined as a two-sided p-value <0.05, and all analyses were performed using Stata package version 12 (Stata Corp., College Station, Texas, 2007).

## Results

During the study period, 310 patients were enrolled, as they were hospitalized with a diagnosis of suspected bacterial infection or sepsis at entry. Of these, 50 patients (16.1%) were excluded from the final sample because of underling conditions potentially associated with immune dysregulation such as AIDS (22), bone marrow transplantation (5), malignancy (13) and other hematologic diseases (10). The final sample therefore included 260 patients, 165 (63.5%) males, mean age 59.1±19.5 years. Sepsis was diagnosed at discharge in 105 patients (40.4%); septic shock in 6 (2.3%); in 60 (57.1%) patients with a diagnosis of sepsis at least 1 microorganism was isolated.

Mean SOFA upon admission turned out 4.03 ± 2.0; only for 4 septic patients it was not possible to retrieve data from medical records. Among patients without sepsis, SOFA scores could be calculated for 43 patients only, being 1.2±1.7. Among patients with sepsis, 41.9% had a qSOFA ≤2; all of these, however, had a SOFA score ≥2, in keeping with the low prevalence of septic shock (5.7%) and low mortality (16%) in our series.

Single positive blood cultures yielding *Staphylococcus hominis*, *Staphylococcus haemolyticus*, *Staphylococcus epidermidis* and *Propionibacterium spp*. were found in 4 patients with infection and were interpreted as contaminants. One hundred seventy-seven (68%) patients had a Charlson Comorbidity Index (CCI) ≤3; 53 (20%) patients a CCI of 4 to 6 and 30 (12%) patients a CCI >6. Mean duration of hospitalization was 11.9±8.3 days. Described in Table 1 are the frequencies of diabetes, renal disease, and in-hospital mortality, whereas sources of infection are shown in Table 2.

**Table 1:** Comparison of clinical and demographic characteristics between patients with infection and sepsis.

Variables	Overall n = 260	Infection n = 155	Sepsis n = 105	*p*
Age, mean (SD), years	59.1 (19.5)	54.9 (18.3)	65.4 (19.6)	<0.001*
Males, n (%)	165 (63.5)	101 (65.1)	64 (60.9)	0.49**
Charlson Comorbidity Index (CCI), mean (SD)	2.7 (3.0)	2.2 (2.6)	3.5 (3.3)	<0.001*
Diabetes, n (%)	55 (21.3)	27 (17.7)	28 (26.7)	0.08**
Septic shock, n (%)			6 (5.7)	
Kidney disease, n (%)	60 (23.1)	20 (12.9)	40 (38.1)	<0.001**
qSOFA 1, n (%)	54 (20.8)	28 (18.1)	26 (24.7)	
qSOFA 2, n (%)	34 (13.1)	6 (3.9)	28 (26.7)	
qSOFA 3, n (%)	7(2.7)	0	7 (6.7)	<0.001**
Positive blood cultures, n (%)	64 (24.6)	4 (2.6)	60 (57.1)	<0.001**
Hospital stay, days, mean (SD)	11.9 (8.3)	10.9 (8.4)	13.4 (8.0)	0.018*
Intra hospital mortality, n (%)	21 (8.1)	4 (2.6)	17 (16.2)	<0.001**

qSOFA, quick Sequential (Sepsis-Related) Organ Failure Assessment.

*t-student test

**χ-square test

**Table 2:** Site of infection in patients with or without sepsis.

Sepsis	n, (%)	Infection only	n, (%)
Lower respiratory tract infection	30 (28.5)	Lower respiratory tract infection	57 (36.7)
Urinary tract infection (UTI)	20 (19.0)	Skin and soft tissue infection	28 (18.0)
Skin and soft tissue infection	11 (10.5)	Spontaneous bacterial peritonitis (SBP)	15 (9.6)
Intra-abdominal infection	10 (9.5)	Enteritis	12 (7.7)
Endocarditis	3 (2.8)	Neuraxis disease	7 (4.5)
Gastroenteritis	3 (2.8)	Porto-systemic encephalopathy	6 (3.9)
Meningoencephalitis	3 (2.8)	Urinary tract infection (UTI)	6 (3.9)
Catheter-related bloodstream infection	2 (1.9)	Pericarditis	5 (3.2)
Osteomyelitis	2 (1.9)	Otomastoiditis	1 (0.6)
Cholangitis	1 (0.9)	Others	18 (11.0)
Pericarditis	1 (0.9)		
Prostatitis	1 (0.9)		
Unknown	18 (17.1)		

**Fig 1:**
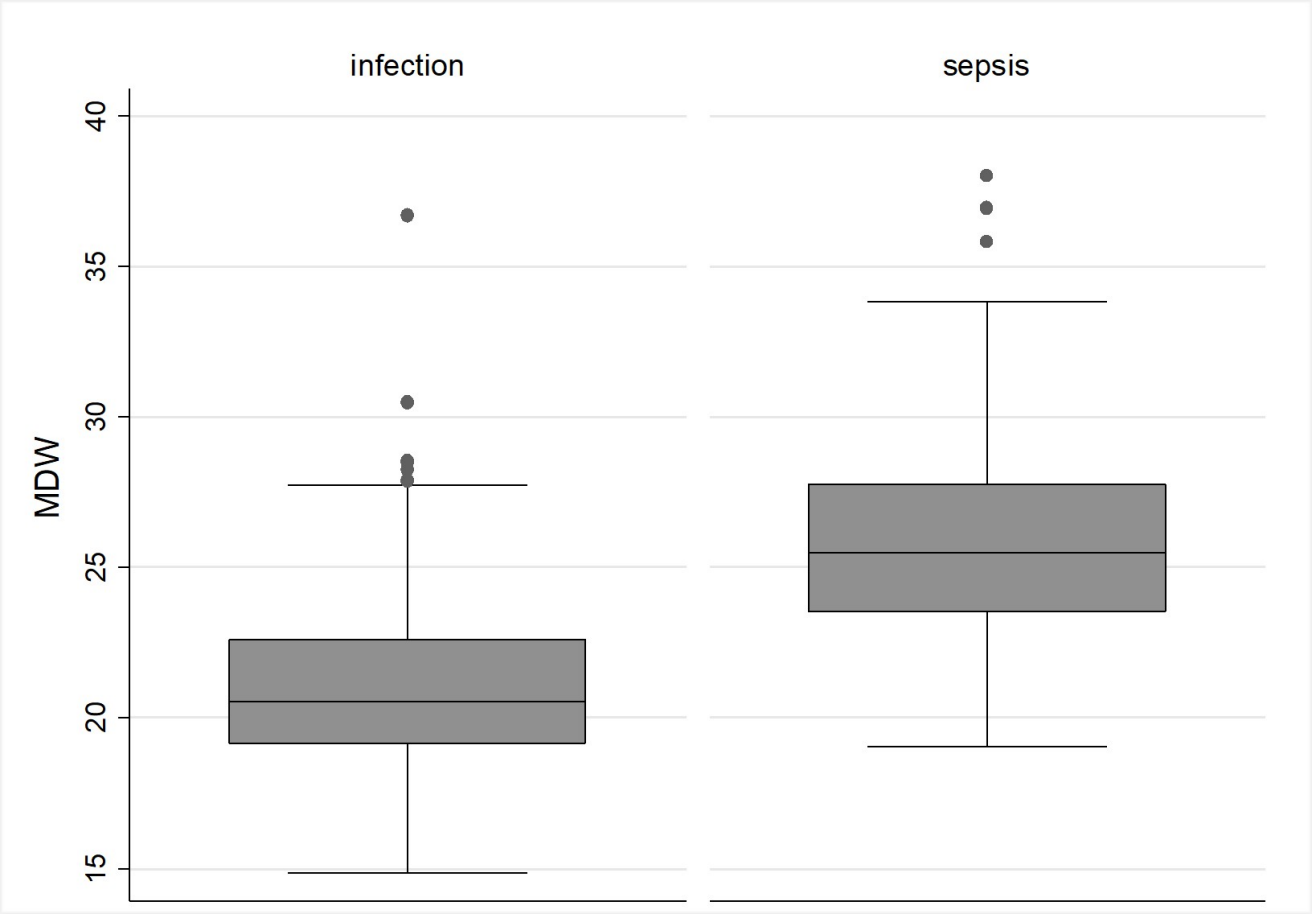
Dot plot analysis of MDW values compared between patients with infection and patients with sepsis.

As showed in Table 1, in our series, sepsis was linked with CCI (3.5±3.3 vs 2.2±2.6, p<0.001), kidney disease (40 patients, 38.1%, vs 20 patients, 12.9%, p<0.001), qSOFA (1.0±0.9 vs 0.2±0.5, p<0.001); days of hospital stay (13.4±8.0 vs 10.9±8.4, p = 0.018) and intra-hospital mortality (17, 16.2%, vs 4, 2.6%, p<0.001). A near significant association was found for diabetes (28, 26.7%, vs 27, 17.7%, p = 0.08, Table 1). All assayed blood cell counts are reported in Table 3. Among cell population data, higher MDW values, 25.5 (23.5–27.8) vs 20.5 (19.1–22.6), p<0.001, were observed in patients with sepsis compared with patients with infection without sepsis (Fig 1). Sepsis was diagnosed in 2 patients only with MDW <20 (1.9%, p<0.001) and in 6 patients with MDW <22 (5.7%, p<0.001, Table 3). Interestingly, all the 60 bacteremic patients had MDW values >20. As a consequence, the negative predictive value of MDW values<20 for bacteremia was 100%. Single serum PCT determinations at hospitalization were available for 217 of the 260 patients in the final sample. Median PCT was 21.0±31.0 ng/mL in patients with sepsis, versus 3.0±12.5 ng/mL among patients with infection without sepsis (p<0.001). After dichotomization, a PCT value >1 ng/mL was significantly associated with sepsis (86, 82.7%, vs 15, 13.3%, p<0.001, Table 4).

**Table 3:** Comparison of blood count parameters between patients with infection and sepsis.

Blood count parameters	Overall n = 260	Infection n = 155	Sepsis n = 105	*p*
WBC *10^3^/μL, median (IQR)	9.0 (6.2–13.4)	7.7 (5.9–11.2)	11.4 (7.9–15.3)	<0.001*
Neutrophils *10^3^/μL, median (IQR)	6.7 (3.9–10.9)	5.3 (3.3–8.3)	9.7 (6.4–12.4)	<0.001*
Lymphocytes *10^3^/μL, median (IQR)	1.2 (0.7–1.7)	1.4 (0.9–1.9)	0.8 (0.5–1.3)	<0.001*
RBC*10^6^/μL, median (IQR)	4.3 (3.8–4.7)	4.4 (3.9–4.8)	4.1 (3.7–4.6)	0.0098*
HGB, median (IQR)	12.2 (10.7–13.8)	13.0 (11.1–14.1)	11.6 (9.9–13.2)	<0.001*
RDW, median (IQR)	14.8 (13.6–17.0)	14.5 (13.4–16.8)	15.5 (14.2–17.7)	<0.001*
PLT *10^3^/μL, median (IQR)	193 (142–260)	211 (162–307)	169 (127–203)	<0.001*
PDW, median (IQR)	17.0 (16.6–17.4)	16.9 (16.5–17.3)	17.2 (16.9–17.6)	<0.001*
MPV, median (IQR)	9.0 (8.3–9.7)	8.8 (7.9–9.3)	9.2 (8.7–10.1)	<0.001*
MNV, median (IQR)	155 (149–162)	152 (148–158)	161 (155–169)	<0.001*
NDW, median (IQR)	19.8 (18.6–21.9)	19.0 (17.9–20.4)	21.4 (19.8–24.0)	<0.001*
MMV, median (IQR)	182 (174–192)	177 (172–186)	191 (183–199)	<0.001*
MDW, median (IQR)	22.5 (20.0–25.3)	20.5 (19.1–22.6)	25.5 (23.5–27.8)	<0.001*
MDW<20, n (%)	67 (25.7)	65 (42.0)	2 (1.9)	<0.001**
MDW<22, n (%)	114 (43.8)	108 (69.7)	6 (5.7)	<0.001**

WBC, White Blood Count; RBC, Red Blood Cell; HGB, Hemoglobin; RDW, Red Blood Cells Distribution Width; PLT, Platelet Count; PDW, Platelet Distribution Width; MPV, Mean Platelet Volume; MNV, Mean Neutrophil Volume; NDW, Neutrophil Distribution Width; MMV, Mean Monocyte Volume; MDW, Monocyte Distribution Width.

*Kruskal-Wallis test

**χ-square test

**Table 4:** Comparison of MDW and PCT values between patients with infection and sepsis.

Variables	N. of patients	Infection	Sepsis	*p*
procalcitonin, mean (SD), ng/mL	217	3.0 (12.5)	21.0 (31.0)	<0.001*
procalcitonin>1, n (%)	217	15/113 (13.3%)	86/104 (82.7%)	<0.001**
MDW >22, n (%)	260	47/155 (30.3%)	99/105 (94.3%)	<0.001**

MDW, Monocyte Distribution Width; PCT, procalcitonin.

*t-student test

**χ-square test

In the multivariate model including all factors associated with sepsis at univariate analyses, MDW as a continuous variable (OR:1.57 for each unit increase; 95%CI: 1.31–1.87, p<0.001), PCT˃1 ng/mL (OR: 48.5; 95%CI: 14.7–160.1, p<0.001) as well as qSOFA (OR: 4.14 for each unit increase; 95%CI: 1.82–9.38, p = 0.001) turned out independently associated with sepsis. No association was found for age (OR: 1.01; 95%CI: 0.98–1.04, p = 0.52), male sex (OR: 0.34; 95%CI: 0.11–1.05, p = 0.06); CCI (OR: 1.04; 95%CI: 0.88–1.23, p = 0.65), and kidney disease (OR: 0.88; 95%CI: 0.27–2.88, p = 0.83, Table 5).

**Table 5:** Final model of logistic regression for independent predictors of sepsis.

Sepsis	Crude OR (95% CI)	*Univariate p*	Adjusted OR (95% CI)	*Multivariate p*
Male sex	0.83 (0.50–1.39)	0.49	0.34 (0.11–1.05)	0.06
Age (1 year increase)	1.03 (1.01–1.04)	<0.001	1.01 (0.98–1.04)	0.52
Kidney Disease	4.15 (2.25–7.67)	<0.001	0.88 (0.27–2.88)	0.83
CCI	1.17 (1.07–1.28)	<0.001	1.04 (0.88–1.23)	0.65
PCT>1 ng/mL	31.21 (14.83–65.68)	<0.001	48.5 (14.7–160.1)	<0.001
MDW (1 unit increase)	1.60 (1.42–1.81)	<0.001	1.57 (1.31–1.87)	<0.001
qSOFA (1 unit increase)	3.40 (2.32–4.99)	<0.001	4.14 (1.82–9.38)	0.001

CCI, Charlson Comorbidity Index; MDW, Monocyte Distribution Width; PCT, Procalcitonin, AUROC 0.96, goodness-of-fit test 0.01

**Fig 2:**
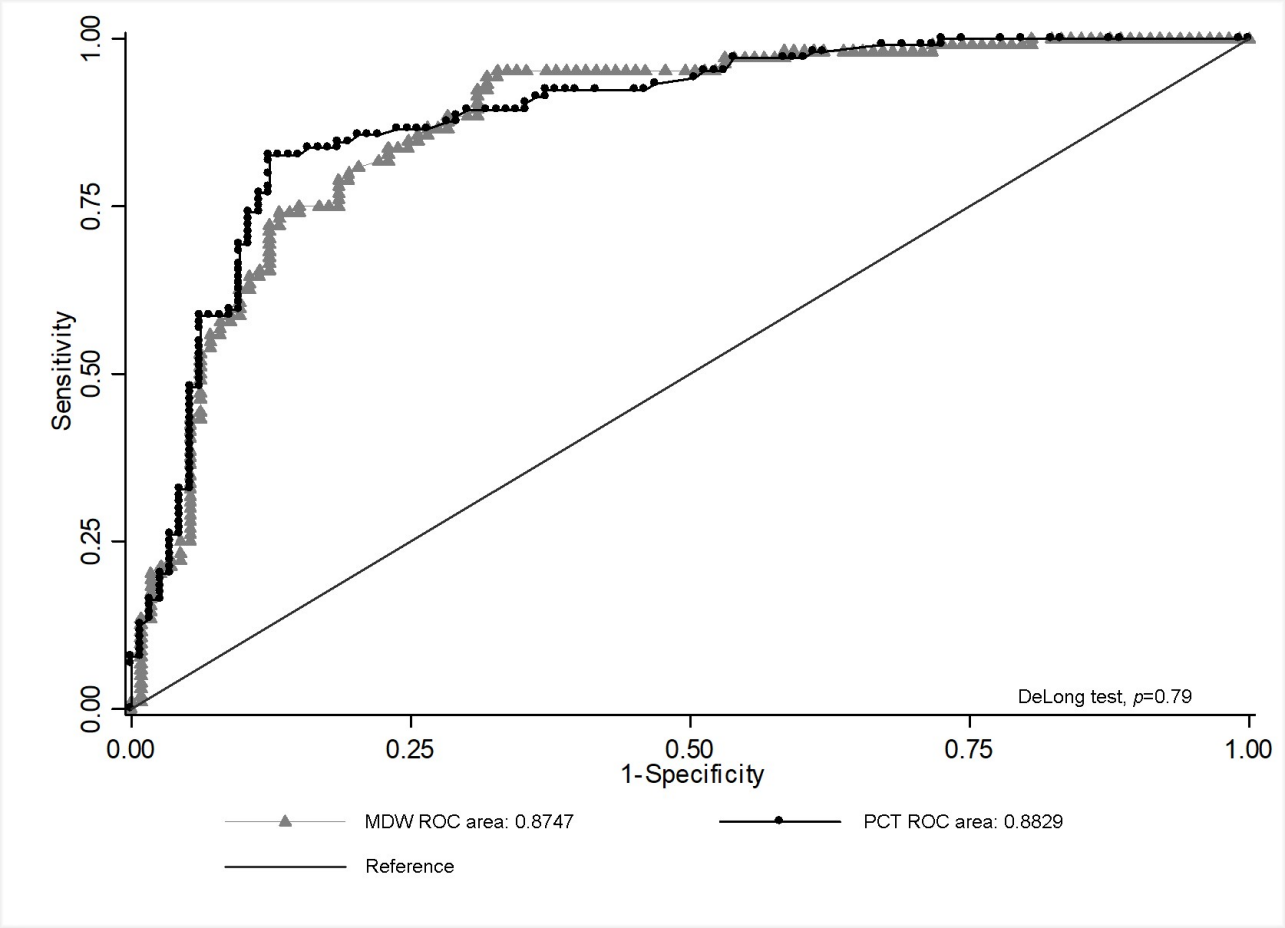
ROC curve analysis for comparison of MDW and PCT in their strength in sepsis prediction. Procalcitonin (●); Monocytes Distribution Width (▲).

At the ROC curve analysis for the prediction of sepsis, the AUC of MDW (0.87; 95%CI: 0.82–0.92) resulted comparable with the AUC of PCT (0.88; 95%CI: 0.84–0.93), with p-values of 0.79 rejecting the hypothesis that the 2 AUCs were significantly different, as shown in Fig 2. Statistical best cut points associated with sepsis were 21.9 for MDW and 1.1 ng/mL for PCT. In Table 6, sensitivity, specificity, PPVs and NPVs with their 95% CI intervals of different cut points for sepsis prediction are showed, both for MDW and PCT.

**Table 6:** Sensitivity, specificity, PPV, PNV of MDW (a) and PCT (b) in predicting sepsis.

a)				
**Variable**	**Sensitivity, % (95%CI)**	**Specificity, % (95%CI)**	**PPV, % (95%CI)**	**NPV, % (95%CI)**
MDW>19	100 (96.5–100)	22.6 (16.3–30.3)	46.7 (40–53.4)	100 (90–100)
MDW>20	98.1 (93.3–99.8)	41.9 (34.1–50.1)	53.4 (46.1–60.6)	97.0 (89.6–99.6)
MDW>21	95.2 (82.2–98.4)	59.4 (51.2–67.2)	61.3 (53.4–68.9)	94.8 (88.4–98.3)
MDW>22	94.3 (88.0–97.9)	69.7 (61.8–76.8)	67.8 (59.6–75.3)	94.7 (88.9–98.0)
MDW>23	81.9 (73.2–88.7)	77.4 (70.0–83.7)	71.1 (62.1–79.0)	86.3 (79.5–91.6)
MDW>24	68.6 (58.8–77.3)	86.5 (80.0–91.4)	77.4 (67.6–85.4)	80.2 (73.4–86.0)
MDW>25	54.3 (44.3–64.0)	91.6 (86.1–95.5)	81.4 (70.3–89.7)	74.7 (67.9–80.7)
MDW>26	41.0 (31.5–51.0)	93.5 (88.5–96.9)	81.1 (68.0–90.6)	70.0 (63.3–76.2)
MDW>27	29.5 (21.0–39.2)	94.2 (89.3–97.3)	77.5 (61.5–89.2)	66.4 (59.7–72.6)
MDW>28	22.9 (15.2–32.1)	96.8 (92.6–98.9)	82.8 (64.2–94.2)	64.9 (58.4–71.1)
MDW>29	18.1 (11.3–26.8)	98.7 (95.4–99.8)	90.5 (69.6–98.8)	64.0 (57.6–70.1)
MDW>30	15.2 (9.0–23.6)	98.7 (95.4–99.8)	88.9(65.3–98.6)	63.2 (56.8–69.3)
MDW>31	13.3 (7.5–21.4)	99.4 (96.5–100)	93.3 (68.1–99.8)	62.9 (56.5–68.9)
b)				
PCT>0.5	85.6 (77.3–91.7)	77.9 (69.1–85.1)	78.1 (69.4–85.3)	85.4 (77.1–91.6)
PCT>1	82.7 (74.0–89.4)	86.7 (79.1–92.4)	85.1 (76.7–91.4)	84.5 (76.6–90.5)
PCT>2	72.1 (62.5–80.5)	89.4 (82.2–94.4)	86.2 (77.1–92.7)	77.7 (69.6–84.5)

## Discussion

The aim of our study was to validate the hypothesis that variations in the spread of monocyte size may be an independent predictor of ensuing sepsis in current clinical practice. Changes in volumetric size of white blood cells are a well-documented consequence of cellular activation upon early infection, as part of innate immunity response [26, 27]. In particular, monocytes are involved in the early response to bacterial invasion of the bloodstream, acting as first interceptors of the invading bacteria, for phagocytosis and further immune processing, with abundant literature evidence of changes in their morphology during infection and sepsis [26, 27]. Monocytes differentiate into amoeboid cells, as assessed by microscopy after Giemsa staining and increased expression of functional markers such as CD16 [28]. As a consequence, the possibility of accurately measuring monocyte size in parallel with routine blood cell counts and indices to monitor ongoing monocyte activation in septic patients is an appealing and potentially high-impact research hypothesis [9, 19, 21] especially in patients and settings whereby monitoring of other biomarkers of sepsis may be expensive, as in resource-poor countries, or more difficult, as in neonates [9, 17, 18, 28]. In line with preliminary studies linking the spread of monocyte width with monocyte immune activation during sepsis [9,17,19], we found that MDW values <20 had a very high NPV for sepsis and a 100% NPV for bacteremias, indicating that low MDW values may be an efficient tool to rule out bloodstream infections. In our series, under a strict statistical point of view a MDW cut off of 21.9 turned out the best threshold for prediction of sepsis. As a consequence, we first chose to dichotomize MDW at >22; this dichotomic variable was both highly sensitive and specific in predicting sepsis. Crouser et al. (2017) first reported that increased MDW values correlated with sepsis in a large sample of patients presenting at an Emergency Department in Ohio [9]; they showed that a value of greater than 20.0 of MDW was effective for sepsis detection based on either Sepsis-2 criteria or Sepsis-3 criteria in a multicenter study enrolling patients from 3 emergency departments [29]. As a consequence, a 2-point difference between their better threshold and ours seems to exist. This could be due to the type and clinical status of hospitalized patients, as well as to technical issues. Among the latter, it is worth pinpointing that we utilized K3EDTA anticoagulant tubes for CBC and MDW determinations, while FDA approved MDW measures using K2EDTA whole-blood venous samples [30]. However, in addition to our best statistical cut-off of 22, we found that a lower cut off of 20 improved the negative predictive value of MDW for sepsis and might therefore be more helpful for real practice purposes.

Crouser et al., 2017, found that MDW was able to discriminate sepsis from SIRS and that the magnitude of MDW elevation correlated with infection severity and organ dysfunction, ranging from low values in patients with limited infections, and increasing in parallel with severity of sepsis. They did not provide, however, a comparison between MDW with other available biomarkers of sepsis as PCT or C-reactive Protein (CRP) [9]. In our study, procalcitonin was prescribed by ward attending physicians for suspected sepsis at patients’ admission, based on medical history and physical examination, in parallel with microbiological assessment. PCT was therefore assayed only in 217 of the 260 consecutive patients, 104 with a diagnosis of sepsis and 113 with a diagnosis of infection without sepsis, allowing us to compare parallel PCT and MDW values by a ROC curve analysis. For the first time—to our best knowledge—in a similar comparison, we found that the AUC of MDW was nearly overlapping with that of PCT, suggesting that MDW may be indeed evaluated further for sepsis prediction in clinical practice [9]. Although sepsis is one of the major cause of mortality for critically ill patients, it is not exclusively restricted to these individuals [31, 32]. Outside of the intensive care units, as in the ED or in lower intensity wards, early diagnosis and rapid treatment initiation are even more crucial for halting progression of disease and poor patient prognosis [1, 2, 33]. The qSOFA and NEWS (National Early Warning Score) scores have been proposed and frequently implemented as a tool to frequently screen patients with infection at risk of organ dysfunction and death [34–37], as widespread and frequent monitoring of biomarkers as PCT may be impractical or too expensive in these settings. Haydar et al. (2017) reported the qSOFA score was efficacious for the identification of patients with sepsis at increased risk of mortality, while performing poorly as a screening tool for early identification of sepsis in the emergency department [33]. In low intensity settings, the use of MDW might therefore become a low-cost alternative to PCT for early detection of ongoing sepsis [9, 29].

There are possible limitations in our study design: first, in spite that our diagnoses of sepsis were based on current Sepsis-3 standards, misclassification of sepsis may have occurred in some cases; this should not limit the relevance of our experimental procedures, however, because of the parallel use of MDW and PCT in the same patients. Second, the study was performed in the setting of real-practice clinical activity, which led to under-prescription of PCT in some patients. Third, host factors, such as HIV infection, ≥2 week steroid or other immune suppressive treatments, such as Hydroxyurea or Methotrexate, and Alcoholism could influence immune cell volumetric evaluation and we had therefore to exclude such patients from our current analyses. The plausibility of the use of MDW in these patients has therefore to wait for further experimental designs. Fourth, and finally, multivariate analyses were not adjusted for cardiovascular diseases, COPD and dyslipidemia. However, both COPD and cardiovascular diseases were considered in the computation of the Charlson Comorbidity Index, that was included as a covariate in multivariate models.

In conclusion, we found that MDW was at least equivalent to PCT in predicting sepsis in patients hospitalized in an Infectious Disease Unit. Additionally, MDW values <20 were invariably associated with negative blood cultures. These results are particularly interesting, as MDW readings will soon be in routine blood counts with next-generation blood cells analyzers. Multicentric investigations to define the real place of this parameter in the timely diagnosis of sepsis are warranted.

## Supporting information

S1 File Minimal database file uploaded as .dta file for STATA 12 analyses. (DTA)
